# Spontaneous bilateral tubal ectopic pregnancy: a gynecological challenge

**DOI:** 10.5935/1518-0557.20230058

**Published:** 2024

**Authors:** Sana Ghades, Abderahmen Daadoucha, Hamed Jemel, Nour Rouis, Mohamed Ridha Fatnassi

**Affiliations:** 1 Department of Gynecology and Obstetrics, University Hospital Ibn El Jazzar, 3100 Kairouan, Tunisia; 2 Department of Radiology, University Hospital Ibn El Jazzar, 3100 Kairouan, Tunisia

**Keywords:** Spontaneous, bilateral, case report, ectopic pregnancy

## Abstract

Bilateral ectopic pregnancy is very rare. Although the frequency of ectopic bilateral pregnancy has increased with the advent of medically assisted procreation, spontaneous bilateral tubal pregnancies remain rare. Early detection of this type of ectopic pregnancy is important to prevent maternal mortality and morbidity. Conservative surgery must also be considered, as preservation of both tubes is presumed to offer better fertility prospects. We report the case of a 35-year-old patient at five weeks of amenorrhea with bilateral ectopic pregnancy diagnosed based on ultrasound scans and confirmed during laparotomy. A 35-year-old woman with a history of three vaginal deliveries, non-smoker, on contraceptives (microprogestins), presented with pelvic pain and amenorrhea of five weeks. A beta HCG test came back positive. Pelvic ultrasound revealed a moderate hemoperitoneum and an empty uterus with hematometra. It also showed heterogeneous left and right adnexal masses measuring 3 cm and 4 cm, respectively. An emergency laparotomy was performed. Per-operatively, two bilateral tubal pregnancies of 3 cm and 4 cm were founded. The patient received conservative treatment with bilateral salpingotomy. Postoperative management was uneventful. The diagnosis of spontaneous bilateral tubal ectopic pregnancy is rare and often established at the time of surgery, hence the importance of a rigorous and vigilant examination of the two tubes during ultrasound examination and surgery, so as not to miss it and to better prevent maternal mortality. Conservative surgery must be carefully chosen.

## INTRODUCTION

Bilateral ectopic pregnancy is very rare. It is estimated to occur in 1/750 to 1/580 ectopic pregnancies (Wakankar & Kedar, 2015). Although the frequency of ectopic bilateral pregnancy has increased with the advent of medically assisted procreation, spontaneous bilateral tubal pregnancies remain rare. Early detection of this type of ectopic pregnancy helps to prevent maternal mortality and morbidity.

We report the case of a 35-year-old patient at five weeks of amenorrhea with bilateral ectopic pregnancy diagnosed based on ultrasound scans and confirmed during laparotomy.

## CASE REPORT

**Figure 1:**
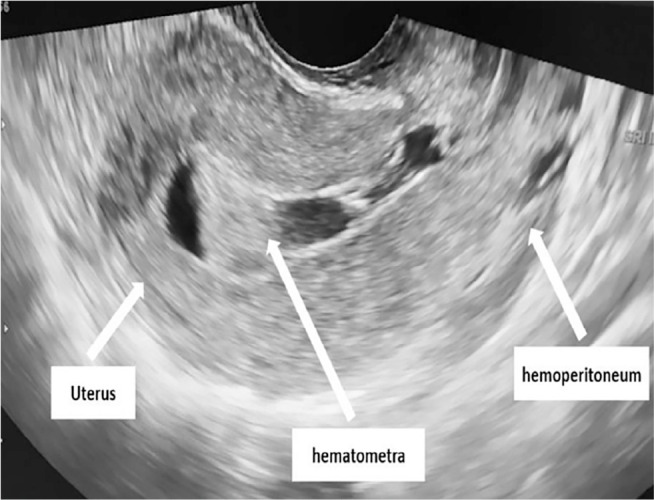
Pelvic ultrasound scan showing a moderate hemoperitoneum and an empty uterus with hematometra.

**Figure 2:**
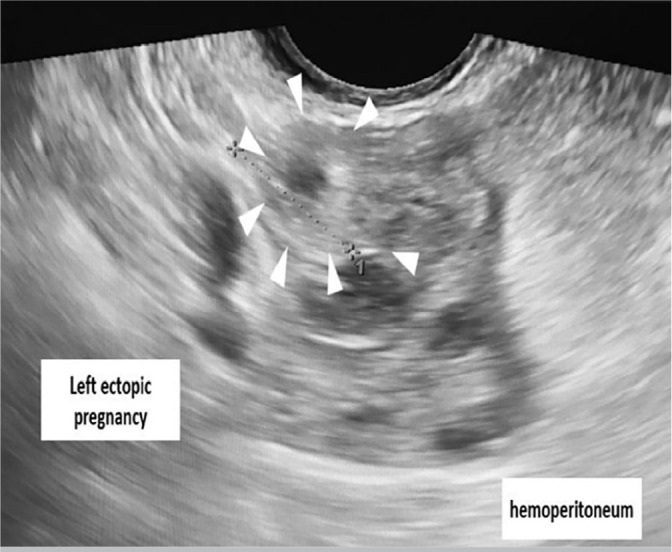
Pelvic ultrasound scan showing right adnexal masses (arrows) measuring 3*4 cm.

**Figure 3:**
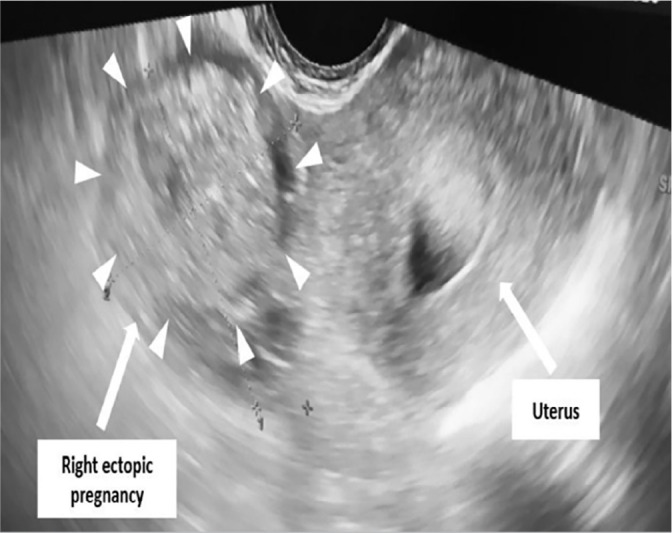
Pelvic ultrasound scan showing right adnexal masses (arrows) measuring 3*4 cm.

**Figure 4:**
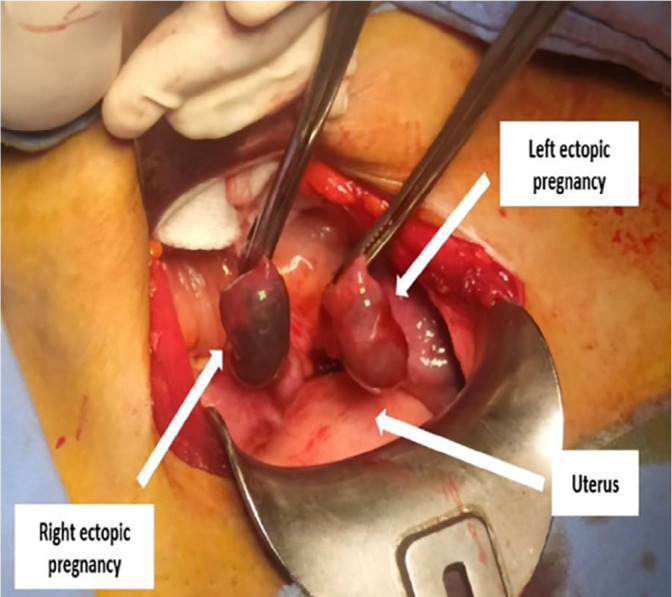
During surgery, two bilateral tubal pregnancies of 3 cm and 4 cm were found.

A 35-year-old woman with a history of three vaginal deliveries, non-smoker, under contraception with microprogestins, presented with pelvic pain and amenorrhea for five weeks. On admission, blood pressure was 10/06 and heart rate was 100 beats/min. The patient had normocolored conjunctivae and tenderness in the left iliac fossa; speculum examination showed a macroscopically healthy cervix with intracavitary bleeding of low abundance; vaginal examination showed a long-closed cervix with tenderness at the posterior cul-de-sac. She had a β HCG level of 4445 mIU/Ml. Pelvic ultrasound scans revealed a moderate hemoperitoneum and an empty uterus with hematometra (Figure 1). It showed also heterogeneous left (Figure 2) and right adnexal masses (Figure 3) measuring 3 cm and 4 cm, respectively. An emergency laparotomy was performed. During surgery, two bilateral tubal pregnancies of 3 cm and 4 cm were found (Figure 4). The rest of the abdomen was without abnormalities. The patient received conservative treatment with a bilateral salpingotomy. Histological results confirmed the diagnosis of bilateral ectopic pregnancy with the presence of placental villi in both fallopian tubes. Postoperative management was uneventful.

## DISCUSSION

Bilateral ectopic pregnancy is primary when it occurs spontaneously, as in the case of our patient, or secondary when it occurs following in vitro fertilization. Bilateral ectopic pregnancy after natural conception is the rarest form of ectopic pregnancy. It occurs in around 1 of 200,000 pregnancies (Wakankar & Kedar, 2015; Chinokwetu-Marere, 2013; Jena *et al*., 2016).

Risk factors for spontaneous bilateral ectopic pregnancy are the same for unilateral ectopic pregnancy, namely: pelvic inflammatory disease, previous ectopic pregnancy, multiple sexual partners, history of infertility, conception after ovulation induction, fallopian tube abnormality, and contraception with microprogestins or IUD. The main risk factor in our patient was contraception with microprogestins. Almost 50% of the patients with an ectopic pregnancy have no risk factors. Ectopic pregnancy should be considered in any patient of childbearing age who consults for pelvic pain even in the absence of risk factors (Mol *et al*., 2014).

More than 95% of ectopic pregnancies occur in the fallopian tubes. The ampulla was the most common site, such as in our case, where there was a bilateral ectopic pregnancy in the ampulla of both fallopian tubes (Mol *et al*., 2014).

The diagnostic criteria for bilateral tubal pregnancy were first defined by Fishback (1939), who suggested that parts of the fetus or the fetus itself should be found with parts of the placenta at two tubes.

Several theories have been proposed to explain the mechanisms of occurrence of bilateral tubal pregnancy. The first theory suggests that bilateral tubal gestation requires multiple ovulations to occur simultaneously, oocytes to be fertilized, and oocytes to implant at sites of tubal damage. The second theory is the transperitoneal migration of trophoblast cells from tube to tube (American College of Obstetricians and Gynecologists, 2008). Multiple fetation, another possible etiology, involves the fertilization and development of a second oocyte when a woman is already pregnant. This situation is considered an extremely rare event in humans and is difficult to prove. Diagnosis is usually suspected when severe growth deficiency is apparent in a multiple pregnancy. Another explanation could be that the second tubal pregnancy occurred after the first disappeared. If the first tubal pregnancy is spontaneously aborted or was in the process of abortion, a second ovulation may have occurred during this period, hence the finding of bilateral tubal pregnancies (American College of Obstetricians and Gynecologists, 2008).

Most patients with bilateral tubal pregnancy present with the same clinical signs as those with unilateral ectopic pregnancy. The most frequent signs are the triad of amenorrhea, metrorrhagia and abdominal pain. Serum β-HCG levels do not correlate with bilateral disease. Ultrasound is not helpful in identifying bilateral ectopic pregnancy. Unilateral ectopic pregnancy has the same clinical presentation as bilateral ectopic pregnancy; therefore, proper ultrasound examination must include both tubes. Some authors have described unintended discovery of cases during laparoscopy (Fishback, 1939; American College of Obstetricians and Gynecologists, 2008; Creanga *et al*., 2011; Chopra *et al*., 2009).

There currently are no specific recommendations for the management of spontaneous bilateral ectopic pregnancies, nor a preference for radical or conservative treatment. Also, few studies evaluated the use of laparoscopy or laparotomy in the treatment of this type of ectopic pregnancy. In general terms, when treating young women who desire to have children, the literature favors conservative treatments devised to preserve fertility (Frates *et al*., 2014; Wang *et al*., 2014; Sentilhes *et al*., 2009). In our case, the patient was hemodynamically stable and desired conservative management; salpingotomy was performed without incidents.

Why is spontaneous bilateral tubal ectopic pregnancy a gynecological challenge? The diagnosis of spontaneous bilateral tubal ectopic pregnancy is rare and often established at the time of surgery. Even though rare, spontaneous bilateral tubal ectopic pregnancy can cause maternal death in the first trimester. This case shows that a rigorous and vigilant examination of the two tubes during ultrasound examination and surgery is necessary to prompt the identification and introduction of interventions to prevent maternal mortality and morbidity. Conservative surgery must also be considered, since preservation of both tubes is presumed to offer better fertility prospects.

## CONCLUSION

The diagnosis of spontaneous bilateral tubal ectopic pregnancy is rare and often established at the time of surgery, hence the importance of a rigorous and vigilant examination of the two tubes during ultrasound examination and surgery so as not to miss it and to better prevent maternal mortality. Conservative surgery must be carefully chosen.
